# Is Further Evaluation of Areas with Faint MDP Uptake Needed in Individuals with Oligo-Metastatic Prostatic Adenocarcinoma?

**DOI:** 10.1055/s-0042-1757281

**Published:** 2022-10-28

**Authors:** Anshul Sharma, Ankur Dwivedi

**Affiliations:** 1 Department of Nuclear Medicine, Homi Bhabha Cancer Hospital and Research Centre, Punjab, India; 2 Department of Radiodiagnosis, Homi Bhabha Cancer Hospital and Research Centre, Punjab, India

**Keywords:** high-risk prostate cancer, ^99m^ Tc-MDP, skeletal scintigraphy, bone marrow metastasis, oligometastatic disease

## Abstract

A 42-year-old male patient with high-risk prostate adenocarcinoma underwent baseline ^99m^ Tc-methylene diphosphonate skeletal scintigraphy, which revealed two skeletal metastases and an area of faint radiotracer uptake in the left femoral shaft. In view of oligo-metastatic nature of the disease in the bone scan and the young age of the patient, he was a candidate for metastases-directed treatment. Single photon emission computed tomography (SPECT)/CT was performed to further characterize this lesion. It was revealed to be a small soft tissue density lesion within the fatty bone marrow density, suggesting bone marrow involvement. A more sensitive evaluation of such areas with faint radiotracer uptake may be needed in high-risk prostate cancer patients where access to advanced modalities is limited. Their significance will also need reassessment as their detection will improve with technological advancements.

## Introduction

Prostatic adenocarcinoma is the second commonest cancer in men. 1 Survival in these patients is a function of metastatic burden, with patients diagnosed at an earlier stage showing longer survival. 2 Optimum treatment and prognostication are dependent on accurate staging. This is especially important in the case of oligo-metastatic disease where more aggressive options are available. 3 4 To this end, the European Association of Urology (EAU) recommends metastatic screening in high-risk patients with localized/locally-advanced disease, 5 which includes cross-sectional imaging and bone scan. Though recent studies have proven prostate specific membrane antigen-positron emission tomography (PSMA-PET/CT) to be superior to a bone scan, particularly in the detection of marrow metastases 6 ; its higher cost and lower availability are significant limitations. This is especially true in low-income countries. Still, a bone scan is not entirely helpless in case of marrow lesions as some reactive uptake is expected in the surrounding regions. With the advancements in image processing, camera technology, and machine learning, such areas with low-intensity uptake will be picked up at a higher rate. The present case is one example of the importance of such low-intensity uptake where access to more advanced modalities was limited.

## Case

**Fig. 1:**
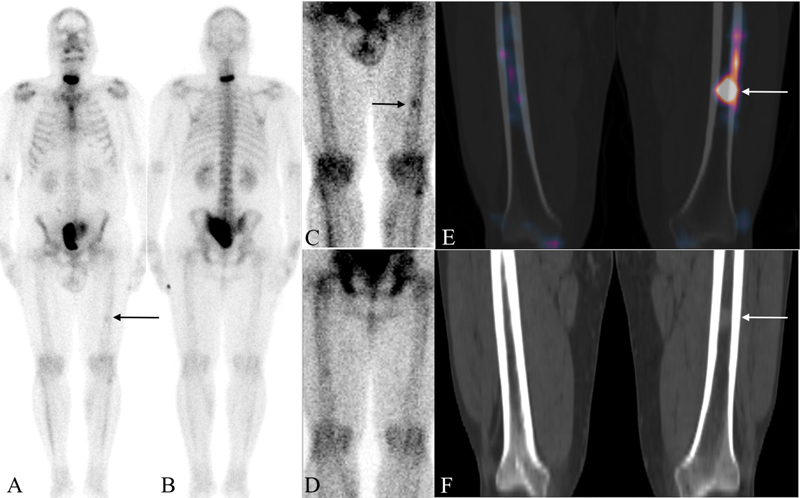
Planar whole body ( **A** [anterior]), ( **B** [posterior]); regional spot (window adjusted; **C** [anterior], **D** [posterior]); SPECT/CT ( **E** ) and CT ( **F** ) images in a 42-year-old male patient with proven prostatic adenocarcinoma, PSA of 102 ng/mL and Gleason score of (5 + 4). Planar images showed an area of faint radiotracer uptake in the left mid-femoral shaft ( **A** , **C** — *arrows* ) and soft tissue density lesion in the corresponding region in SPECT/CT ( **E** , **F** — *arrows* ).

A 42-year-old male patient with recently diagnosed prostate adenocarcinoma was referred to the department of Nuclear Medicine for baseline ^99m^ Tc-methylene diphosphonate skeletal scintigraphy ( ^99m^ Tc-MDP). Gleason score was (5 + 4) and serum PSA was 102 ng/mL. The patient was found to have locally advanced disease in magnetic resonance imaging (MRI). Planar ^99m^ Tc-MDP scintigraphy revealed intense radiotracer uptake in the C6–7 vertebral region and the sacrum, which was indicative of osteoblastic skeletal metastases. Thus, the patient was categorized as having oligo-metastatic disease and a candidate for metastases-directed therapy. In addition to these findings, an area of faint radiotracer was noted in the mid-shaft region of the left femur ( Figs. 1A , **B** and enhancement with gamma correction **C** , **D** ) *.* Due to the oligo-metastatic nature of the disease, the reporting nuclear medicine physician decided to confirm his suspicion of suspected bone marrow involvement. However, the patient could not undergo a repeat MRI (of the thigh) or ^68^ GaPSMA PET/CT due to financial reasons. Therefore, regional single photon emission computed tomography (SPECT)/CT of the thigh was acquired, which confirmed increased radiotracer uptake in the left mid-femoral region ( Fig. 1E ). A small soft tissue density lesion within the bone marrow was noted in the corresponding CT images ( Fig. 1F ). This lesion was surrounded by the fatty density. The two bony lesions and one marrow lesion, along with the young age of the patient, allowed the patient to avail metastases-directed therapy to improve his survival and prognosis.

## Discussion

The European Association of Urology recommends metastatic screening with cross-sectional imaging and skeletal scintigraphy in only those patients, who present with high-risk localized/locally advanced prostatic adenocarcinoma. 5 Increasingly ^68^ Ga-PSMA (prostate-specific membrane antigen) PET (positron emission tomography)/CT has been filling in the role of cross-sectional imaging. 7 Recent studies have shown it to be comparable to skeletal scintigraphy in the detection of skeletal metastases 6 and superior to the latter in the detection of marrow and osteolytic lesions. 8 With increasing importance being given to the diagnosis and aggressive treatment of oligo-metastatic prostatic cancer, correct identification of all metastatic sites has become important and PSMA PET/CT has become the preferred modality. 3 Despite the fact that the best possible modality should be used to correctly identify all lesions before opting for aggressive treatment of oligometastatic disease, the limited availability and higher cost of PSMA PET/CT means that many centers continue to use the combination of regional cross-sectional imaging and skeletal scintigraphy for baseline staging. Therefore, in this limited scenario, as seen in the presented case, we may need to attach greater significance to the areas of faint radiotracer uptake in baseline skeletal scintigraphy. While marrow lesions by themselves do not show increased uptake on the bone scan, there are reactive changes in the surrounding bone, which may be picked up. In our opinion, with the advancements in image post-processing, camera technology, and machine learning, such areas with low-intensity uptake are expected to further increase in number. Even a simple act of changing the gamma, as seen in Fig. 1C , **D** can help us to better delineate this lesion. 9 The issue of false positives can be addressed with the judicious use of SPECT/CT while taking note of the impact on patient management. Judicious use of SPECT/CT incorporates disease stage, risk classification, and serum PSA, which can aid calculation of pre-test probability of finding distant metastases and consequently a need for such sensitive analysis. The drawback of this approach in the discussed case was the likelihood of missing other lesions, but the decision to offer aggressive treatment was influenced by the age of the patient, the locally advanced but operable primary disease, and restricted access to advanced diagnostic modalities.

Finally, we must emphasize that we are not recommending this approach to replace the need for the best available modality, that is, PSMA-PET/CT or MRI. Instead, we are trying to show that with technical improvements, we will be able to see a higher number of such lesions and therefore we will need to address the significance we assign to them. Future studies are needed to address these questions.

## Conclusion

In patients undergoing a bone scan, areas with low avidity should be carefully examined in the context of their likely impact on patient management. With the advancements in image processing, camera technology, and machine learning, such areas with low- and very low-intensity uptake will be picked up at a higher rate and therefore their significance and drawbacks will need to be reassessed, especially in the context of restricted availability of other advanced modalities.
